# Servant leadership and volunteer work engagement in blood donation services: the serial mediating roles of mission valence and volunteer motivation with the moderating role of team atmosphere

**DOI:** 10.3389/fpsyg.2026.1750523

**Published:** 2026-03-25

**Authors:** YingYing Xie, ChengRui Ye, LiHua Yao

**Affiliations:** School of Public Health, Chongqing Medical University, Chongqing, China

**Keywords:** blood donation service volunteers, mission valence, servant leadership, volunteer motivation, work engagement

## Abstract

**Objective:**

To examine the relationship between servant leadership and work engagement among blood donation service volunteers, the mediating roles of mission valence and volunteer motivation, and the moderating role of team atmosphere, which may inform strategies for enhancing work engagement among blood donation service volunteers.

**Methods:**

The Servant Leadership Scale, Volunteer Motivation Scale, Mission Valence Scale, Team Atmosphere Scale, and Work Engagement Scale were used to investigate 1,330 blood donation service volunteers.

**Results:**

① Significant positive correlations were found between servant leadership, mission valence, volunteer motivation, work engagement, and team atmosphere (*r* = 0.552, 0.686, 0.61, 0.621, 0.615, 0.767, 0.652, 0.562, 0.774, and 0.578, *p* < 0.01); ② Servant leadership was positively associated with volunteer work engagement in blood donation services (*β* = 0.595, *p* < 0.01) with mission valence and volunteer motivation playing separate mediating roles (*β* = 0.053, 0.295, *p* < 0.01) and a serial mediating role (*β* = 0.11, *p* < 0.01); ③ Team atmosphere played a moderating role in the serial mediation effect of servant leadership on the work engagement of blood donation service volunteers (*β* = 0.075, *p* < 0.01).

**Conclusion:**

Servant leadership is associated with higher work engagement levels among blood donation service volunteers. It is also associated with work engagement through mission valence and volunteer motivation. Team atmosphere significantly moderates the relationship between mission valence and volunteer motivation, with the association strengthening as team atmosphere increases.

## 1 Introduction

In an era of rapid advancement in healthcare, blood serves as a vital foundation and a critical component in medical treatment. Voluntary blood donation remains the primary source of blood for blood banks (Guo and Yue, 2025). According to data from the World Health Organization, China’s blood donation rate in 2018 was 11.2‰ (People's Government of the People's Republic of China, 2018), which showed a significant gap compared to the rates in high-income countries (31.5‰) (World Health Organisation, 2023). Meanwhile, statistics from China’s National Health Commission indicate that the national blood donation rate increased 12.2‰ in 2023 (National Health Commission of the People's Republic of China, 2023) but declined to 11.4‰ in 2024 (National Health Commission of the People's Republic of China, 2025), highlighting the persistent imbalance between blood supply and demand. To address the critical shortage of blood supplies and the imbalance between demand and supply in China, encouraging public participation in voluntary blood donation and promoting the development of voluntary blood donation have become key priorities in the health sector. Blood donation service volunteers play a pivotal role in this endeavor.

Blood donation service volunteers steadfastly fulfill their responsibilities of “promotion, service, donation, and recruitment” during their service. They collaborate with blood center staff, with each performing their respective duties, to jointly advance the development of the blood donation cause (Yang et al., 2020). Previous studies have clearly demonstrated that volunteer blood donation services are crucial for the long-term sustainability of voluntary blood donation. Strengthening the management of volunteer blood donation teams can enhance service quality and improve donor satisfaction (Zhou et al., 2022; Yu and Li, 2018). The Chinese government emphasizes in its guidance on promoting voluntary blood donation that “efforts should be strengthened to enhance voluntary blood donation services and reinforce the development and management of volunteer teams,” highlighting the pivotal role of volunteers in advancing the cause of voluntary blood donation (Red Cross Society of China, 2022).

However, in recent years, the global community has faced a significant decline in the number of volunteers and a severe attrition rate among them. Volunteers’ willingness to continue serving, their participation levels, and retention rates have all decreased (Forner et al., 2023; Tsai et al., 2023). China also faces challenges such as insufficient volunteer numbers and high attrition rates (Jiang, 2019). Over time, individuals within volunteer organizations gradually decrease the frequency and number of volunteer activities they participate in until they cease involvement altogether. Consequently, while the total membership of volunteer organizations may not have decreased, the actual number of individuals actively participating in volunteer activities steadily declines (Jia, 2017). Moreover, as volunteer organizations operate on a public welfare basis and lack incentive mechanisms, retaining occasional volunteers over the long term is difficult, which hinders volunteer retention and the provision of ongoing volunteer services (Heetae et al., 2020). During volunteer service, some volunteers may display lower levels of engagement and motivation, resulting in shirking responsibilities in volunteer service work (Freeman, 1996). The intensifying volunteer attrition, declining willingness to volunteer, and reduced commitment to volunteer work all hinder efforts to enhance volunteer engagement. China’s voluntary blood donation services encounter similar challenges. Although the registered volunteer workforce is large, the number of volunteers actively participating and making effective contributions remains low. The insufficient commitment to volunteer service hinders the sustainable development of the voluntary blood donation cause. Therefore, it is crucial to explore the pathways influencing volunteer work engagement in blood donation services and enhance the efficiency and quality of volunteer services.

Previous studies have found that servant leadership and high levels of volunteer motivation can effectively enhance work engagement, but the specific pathways and mechanisms underlying this enhancement have remained insufficiently explored. Domestic research has primarily focused on exploring management strategies for volunteer teams at the organizational level, with few studies examining relevant mechanisms from the perspectives of volunteers’ perceptions of leadership styles, mission valence, and motivation, which remain relatively scarce. Research on volunteers has primarily focused on groups such as community and sports event volunteers, with limited studies on blood donation service volunteers. Given the medical nature of the working environment for blood donation service volunteers, they are required to possess higher levels of professional expertise, the ability to provide emotional reassurance to donors, and the capacity for sustained and stable service within the blood donation team. In contrast, community or sports volunteers typically engage in daily social interactions and organizing activities, representing a significant difference from the duties of blood donation service volunteers. Consequently, findings from research on community or sports volunteers may not be directly applicable to blood donation service volunteers. However, research on the pathways and mechanisms influencing the work engagement of blood donation service volunteers is scarce. Therefore, this study employs social exchange theory as its primary theoretical framework, supplemented by self-determination theory, the ability-motivation-opportunity theory (AMO), and social identity theory, to examine the influence of leadership style, mission valence, volunteer motivation, and team atmosphere on volunteer work engagement within blood donation services. The aim is to identify the pathway mechanisms influencing volunteer commitment to blood donation services, thus providing an evidence base for the development of subsequent intervention measures.

## 2 Theoretical framework and formulation of research hypotheses

### 2.1 Theoretical framework

Social exchange theory provides a systematic framework for understanding the impact of servant leadership on the work engagement of blood donation service volunteers and their underlying mechanisms. This theory posits that social exchange constitutes a voluntary act whereby, when one party extends assistance or favors to another, the giver anticipates future reciprocation. This anticipated reciprocation is founded upon the belief that the recipient will render fair compensation over the long term (Blau, 1963). When individuals anticipate gaining benefits, they are likely to offer assistance; however, when they foresee excessive costs, they may decline to help. Among these, the principle of reciprocity stands as a crucial rule and norm within social exchange, fostering trust, commitment, and cooperation to strengthen social bonds (Cropanzano and Mitchell, 2005). Thus, successful reciprocal exchanges can transform transactional relationships into higher-quality social exchanges beyond mere economic transactions (Blau, 1986). Blau also noted that emotional rewards can be gained through social exchange, such as satisfaction, intimacy, or emotional support derived from interpersonal relationships themselves. Simultaneously, emotions play a crucial role in social exchange processes, as they can influence exchange partners’ perceptions and affect the likelihood of future exchanges (Lawler and Thye, 1999). If individuals experience positive emotions within the relationship, they are more likely to engage in favorable behaviors; conversely, when individuals experience negative emotions within the relationship, they are more likely to exhibit unfavorable behaviors.

**Figure 1:**
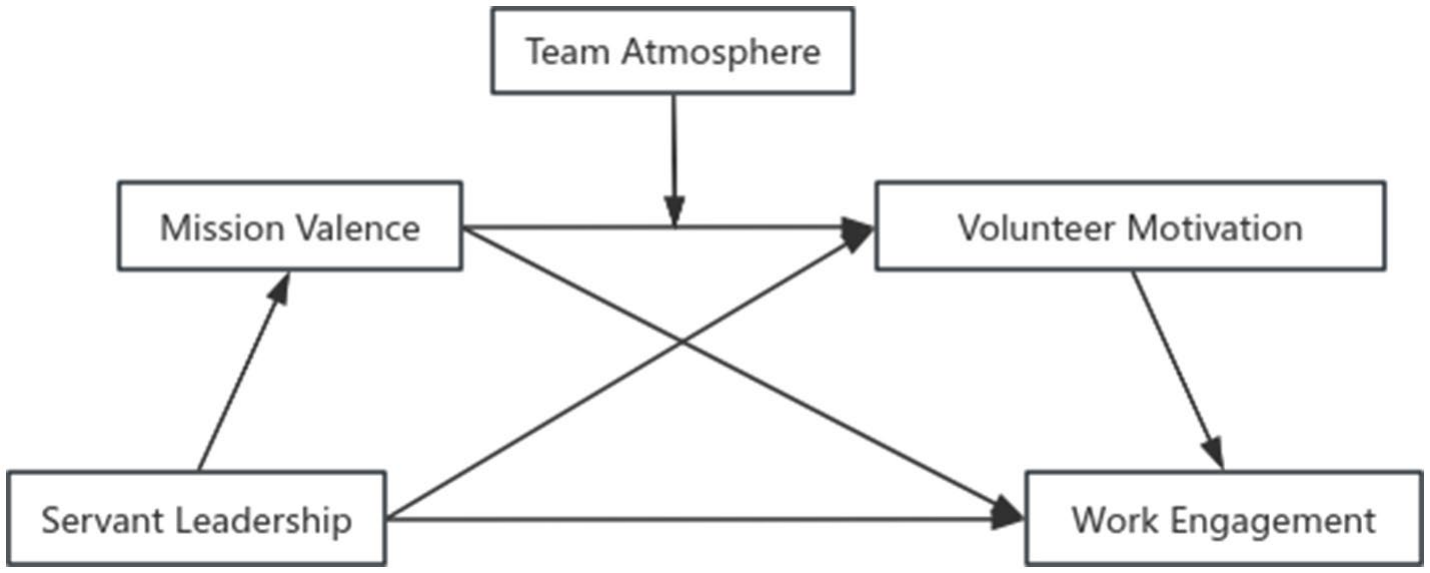
Theoretical hypothesis model.

Social exchange theory has been extensively discussed within corporate settings, yet its relationship on volunteers’ work engagement remains underexplored. Based on the principle of reciprocity, the care, support and assistance provided by servant leadership to blood donation service volunteers may constitute a form of “giving.” This giving may foster a sense of obligation among volunteers to “repay” the favor. The most direct form of repayment for volunteers may be to reciprocate with heightened enthusiasm and engagement in their work, potentially establishing a stable social exchange relationship. Servant leadership may provide instrumental support to volunteers and foster emotional fulfillment and closeness through respect and care. This intrinsic emotional benefit may correlate with heightened mission efficacy and volunteer motivation, potentially reinforcing volunteers’ willingness to engage in sustained “exchange” through continued commitment. Meanwhile, team atmosphere may plays a significant role in this process. A positive team atmosphere may foster a pleasant emotional experience, potentially leading volunteers to recognize and value the mission of volunteering more highly. This may be associated with strengthening the psychological process whereby value recognition is transformed into volunteer motivation. Conversely, a negative team atmosphere may be associated with weakening this process. Within the systematic framework of social exchange theory, to explore the operational mechanisms of serial mediation and moderation effects in this study, we additionally incorporate self-determination theory, AMO theory and social identity theory as complementary perspectives. These assist in understanding how servant leadership, by fulfilling volunteers’ psychological needs during the exchange process, may trigger their intrinsic psychological transformation. As shown in Figure 1.

### 2.2 Formulation of research hypotheses

#### 2.2.1 Servant leadership and work engagement

Work engagement refers to the process by which organizational members integrate their personal identity with their occupational roles through self-management. When work engagement is high, individuals are likely to devote their energies to role-related behaviors, and when work engagement is low, individuals may disengage their personal identity from their occupational roles (Kahn, 1990). Research indicates that volunteers with higher levels of work engagement are associated with a deeper understanding of and identification with the organization’s goals, mission, and values, and may be more inclined to actively participate in volunteer activities (Tian et al., 2024). Servant leadership refers to leaders who prioritize the needs, aspirations, and interests of others over their own, with primary motivation being to serve others rather than to lead and control them (Greenleaf, 2007). Organizations that adopt a servant leadership style in management may be associated with enhanced employee job satisfaction, performance, engagement, and work quality (Nourhan et al., 2025). Social exchange theory posits that reciprocal voluntary reciprocity exists within social relationships. When one party provides support or resources to another, the recipient may acquire an obligation to reciprocate. Thus, the social exchange process is fundamentally a relationship where two or more actors achieve mutual benefit through the voluntary transfer of certain resources (Vladimir, 2014). The leader-member exchange concept within social exchange theory further posits that leaders establish high-quality exchange relationships with trusted partners—interactive bonds characterized by mutual trust, respect, attraction, and influence (Dansereau et al., 1975). The servant leadership style characterized by sincerity, fostering member growth, and demonstrating respect and care helps establish long-term, mutually beneficial relationships with organizational members. This approach is likely to be associated with enhanced members’ work efficiency, commitment, and job satisfaction (Algarni and Munshi, 2023; Mohammad et al., 2024). This intimacy may encourage servant leadership to provide greater assistance and support to members, helping them achieve success by treating organizational members as equals, meeting their needs, and granting them appropriate autonomy. When members strongly perceive support and assistance from servant leadership, it is likely to be associated with their reciprocating with positive attitudes and behaviors, spontaneously repaying servant leadership through heightened levels of work engagement. Based on the above analysis, our study proposes the following hypothesis:

*H1:* Servant leadership positively relates to work engagement among blood donation service volunteers.

#### 2.2.2 The mediating role of mission valence

Mission valence refers to the extent to which employees are attracted to and inspired by the organization’s mission and values, thereby proactively integrating themselves into the organizational mission and participating in organizational activities to pursue the achievement of organizational goals and the realization of organizational values (Yu and Zhang, 2023). The identity regulation theory within self-determination theory posits that when individuals fully recognize the importance of a certain behavior to themselves and can identify with these rules, they may experience greater freedom and self-will during the behavioral process, free from pressure and control (Zhao et al., 2016). In such circumstances, personal will can drive the emergence of autonomous behavior. This state is precisely the crucial psychological manifestation of high-quality social exchange. Specifically, the higher an individual’s sense of mission and value toward the organization or the work itself, and the more they perceive the work’s mission as significant, the more they may be motivated to take action, This suggests that the exchange extends beyond mere material transactions to foster a deeper reciprocal relationship based on shared values. Individuals with a strong sense of mission may be more likely to experience a sense of responsibility and meaning in their work, demonstrating heightened motivation and enthusiasm, and exhibiting a more proactive and engaged attitude toward their duties (Lin et al., 2025). Mission valence, as a powerful intrinsic motivator, is likely to be associated with unleashing individuals’ potential and work enthusiasm, enhancing their recognition of their work, potentially leading them to become more focused and committed to their tasks (Liu Z. et al., 2024). Servant leadership emphasizes prioritizing others over self-interest and valuing employees’ personal development and growth. This people-centered leadership style may foster a “service-oriented atmosphere” within organizations, characterized by support, assistance, and encouragement. Under the influence of this atmosphere, organizational members may gain a deeper understanding of the organization’s mission and vision (Liu X. et al., 2024). Simultaneously, given the significance of intrinsic rewards within social exchange theory, servant leadership may foster a profound sense of belonging, engagement, and value among organizational members by communicating organizational goals, providing support, and motivating individuals. This may enable members to deeply appreciate the significance of the organizational mission, aligning it with their personal values. Consequently, members may commit wholeheartedly to their work as an expression of their personal will and as a form of reciprocity toward leaders and the organization (Kimakwa et al., 2021). Based on the above analysis, our study proposes the following hypothesis:

*H2:* Mission valence mediates the relationship between servant leadership and volunteer work engagement in blood donation services.

#### 2.2.3 The mediating role of volunteer motivation

Volunteer motivation, as an intrinsic driving force, may enable individuals to overcome obstacles and engage in volunteer service (Clary et al., 1992). Specifically, individuals with high volunteer motivation may demonstrate a more tangible willingness to serve the public. When their volunteer work positively impacts the public and society, they may experience a sense of pride and accomplishment, and may become more dedicated, energetic, and fully committed to their volunteer service (Mreydem et al., 2025). Previous studies have demonstrated that volunteer motivation, as a positive factor, may be associated with enhancing individuals to enhance their work engagement, satisfaction, and commitment (Mohsin et al., 2023; Dan and Hung, 2022; Ma et al., 2023). Self-determination theory views human motivation as a dynamic continuum, progressing from extrinsic incentives toward intrinsic motivation. It posits that self-determined behavior may originate from environmental inputs and the individual’s needs structure, emphasizing the interaction between personal requirements and the surrounding environment (Vallerand, 2000). Intrinsic motivation is likely to be associated with a positive influence on work engagement (Haivas et al., 2013). Servant leadership is regarded as a positive resource that may foster intrinsic motivation within individuals. Its fundamental pursuit may lie in meeting members’ needs, promoting their personal development, and offering respect and support. Servant leadership tends to emphasize the significance of employees’ work, encouraging subordinates to actively explore the deeper value of volunteer service (김선희 et al., 2018), potentially stimulating their intrinsic motivation to volunteer. According to social exchange theory, stable reciprocal exchanges are associated with trust, commitment, and cooperation between parties, which could potentially lead to more enduring exchange relationships. In other words, servant leadership within organizations may inspire individuals to engage in volunteer service by setting an example, consistently communicating the importance of the organization’s vision and values, and providing the necessary support to meet individual needs. This approach may fuel the driving force behind voluntary participation, potentially motivating individuals to act autonomously and commit to volunteer work with increased focus and responsibility. Based on the above analysis, our study proposes the following hypothesis:

*H3:* Volunteer motivation mediates the relationship between servant leadership and volunteer work engagement in blood donation services.

#### 2.2.4 The mediating role of serial effects on volunteer motivation and mission valence

Moreover, the association of volunteer motivation and mission valence on work engagement is interdependent. The preceding discussion, grounded in the social exchange theory, established the logical foundation for how servant leadership, through resource and support provision, stimulates employees’ sense of reciprocal obligation. However, AMO theory suggests that translating this reciprocation motivation into actual work engagement is not a function of any single factor, but rather emerges from the synergistic interplay of ability, motivation, and opportunity (BosNehles et al., 2023). Here, ability refers to the psychological and cognitive capacities that enable people to effectively engage in an activity; motivation denotes the psychological and emotional tendencies that influence people’s engagement in an activity; and opportunity signifies external environmental factors, such as events or people that either facilitate or impede people’s behavior. Blumberg suggests that employee behavior and performance are influenced by a combination of ability, motivation, and opportunity (Blumberg and Pringle, 1982). Servant leadership, as an external factor, may create supportive environments and resources that provide individuals with valuable opportunities to fulfill their needs and achieve their personal value. When people possess a higher level of mission valence, it is likely to be associated with a stronger capacity to comprehend and internalize the organizational mission and vision, which makes it easier to integrate these into personal objectives. Volunteer motivation may serve as the direct driving force that prompts people to engage in volunteer activities and may be associated with enhancing their commitment to volunteer work. Empirical research suggests a significant positive correlation between mission valence and volunteer motivation. As a positive psychological resource, mission valence may enhance individuals’ identification with the mission of volunteer service, potentially fostering and stimulating their motivation to volunteer (Caillier, 2015; Yuan et al., 2024). In accordance with the principle of reciprocity, servant leadership may prioritize subordinates’ development by providing resources and support. By emphasizing the collective mission and altruistic behavior, it may enhance employees’ identification with the mission’s value. When people perceive the mission as meaningful, the effect of volunteer motivation may be amplified, potentially leading to spontaneous and proactive engagement in work. Based on the above analysis, our study proposes the following hypothesis:

*H4:* Mission valence and volunteer motivation play a serial mediating role in the relationship between servant leadership and volunteer work engagement within blood donation services.

#### 2.2.5 The moderating role of team atmosphere

Social exchange theory emphasizes the importance of emotions in the exchange process. When individuals derive positive emotions from the relationship, they may be more likely to engage in favorable behavior. Team members may adjust their attitudes and behaviors in response to signals within the environment (Anderson and West, 1994). When the team atmosphere aligns with members’ aspirations and is associated with positive impacts on their physical and mental well-being as well as task completion, people may be more likely to remain in the team. They may develop a stronger sense of the team’s mission and vision, which could strengthen the connection between motivation and job identification, potentially motivating individuals to engage more proactively in their work (Chen et al., 2024). This exchange relationship, driven by positive emotions, may further trigger an individual’s identification mechanism. Social identity theory suggests that social identity primarily arises from group membership. People naturally align themselves with groups that share similar characteristics through self-perception and recognition of other members, and as a result, they tend to adopt behaviors that are consistent with those of the group. People may strive to attain or maintain a positive social identity to enhance their self-esteem, self-confidence, sense of belonging, and security (Zhang and Zo, 2006). When individuals operate within a supportive team atmosphere, they may experience a heightened sense of mission in their work, potentially strengthening the impact on performance (Ping et al., 2018). Simultaneously, perceiving a higher level of team atmosphere may enhance the relationship between intrinsic or extrinsic motivation, work engagement and commitment (Xing et al., 2023). The better the team atmosphere people experience, the more they may feel joyful emotions and the greater their identification with team goals. Members may exhibit higher levels of recognition and satisfaction with their volunteer service organization and work. They may be more likely to psychologically identify as internal members of the organization, viewing the organization’s mission as an extension of their personal values, which could activate intrinsic motivation (Dong et al., 2021; Ferdinando and Salvatore, 2023; Yvonne et al., 2023). Research suggests that a harmonious and equitable team atmosphere may help members understand and embrace the team’s mission, potentially internalizing it as their own sense of purpose. This may enhance individual psychological safety and intrinsic motivation, potentially increasing work engagement and further improving team performance (Ziqing et al., 2022; Zhang et al., 2014; Liu et al., 2011). Based on the above analysis, our study proposes the following hypothesis:

*H5:* Team atmosphere moderates the relationship between mission valence and volunteer motivation, with the positive relationship is stronger when team atmosphere is high.

## 3 Objects and methods

### 3.1 Object

This study employed convenience sampling to select members from blood donation service volunteer teams at blood centers and universities for investigation. A total of 1,570 questionnaires were distributed. After excluding invalid responses, 1,330 participants were ultimately included in the study sample, yielding an effective response rate of 84.71%. A total of 111 blood donation service volunteers held a secondary vocational school diploma or high school diploma and below (8.4%), 422 held an associate degree (31.7%), 790 held a bachelor’s degree (59.4%), and 7 held a master’s degree (0.5%); A total of 828 volunteers (62.3%) participated in blood donation services ≤ once per week, 364 volunteers (27.4%) participated twice per week, and 138 volunteers (10.4%) participated ≥ three times per week; A total of 769 blood donation volunteers (57.8%) had served for less than 1 year, 404 (30.4%) had served for 1 to 3 years, 75 (5.6%) had served for 3 to 5 years, and 82 (6.2%) had served for more than 5 years. This study was approved by the Medical Research Ethics Committee of Chongqing Medical University.

### 3.2 Methods

#### 3.2.1 Servant leadership scale

The servant leadership scale developed by Liden et al. (2015) comprises seven items, measuring the following dimensions: conceptual skills, helping employees develop and succeed, emotional support, creating value for the community, putting employees first, empowerment, and ethical behavior. The Servant Leadership Scale has been extensively applied in China. After its initial translation into Chinese by domestic scholars, the scale underwent rigorous proofreading by English language specialists. Through iterative revisions and refinements, the Chinese version was finalized to preserve the original scale’s semantic integrity aligning it with linguistic conventions and avoiding ambiguities. Subsequent validation among corporate employees demonstrated that the Chinese Servant Leadership Scale exhibits strong reliability and validity (Zhang, 2022). Moreover, this study defines “leader” within the team atmosphere questionnaire as “volunteer team leader” to align with the research context. Specific items include: “My volunteer team leader emphasizes the importance of giving back to the community,” “My volunteer team leader puts my best interests ahead of his/her own,” “My volunteer team leader makes my career development a priority,” among others. The Likert 5-point scale was used, with higher scores indicating that participants perceived a greater degree of servant leadership. In this study, we conducted reliability and validity tests on the Servant Leadership Scale. The results showed that the Cronbach’s *α* coefficient for the scale was 0.836, the composite reliability (CR) was 0.85, and the average variance extracted (AVE) was 0.5. These results demonstrate that the Servant Leadership Scale possesses good reliability and validity in this study.

#### 3.2.2 Mission valence scale

This study used the scale developed by Wright and Pandey, comprising three items rated on a 5-point Likert scale (Caillier, 2016). Chinese scholars revised the Mission Valence Scale for its Chinese version. This process began with a team comprising English-proficient professionals, public administration experts, and human resource management specialists who jointly assessed the scale’s applicability, adjustments, and translation. Extensive interviews were conducted with research participants to ensure the questionnaire items align with practical Chinese contexts and reflect domestic realities. This led to the development of a Chinese-language Mission Valence Scale tailored to the Chinese context (Lin and Chen, 2018). Moreover, this study defines “organization” as “volunteer service team” within the Mission Valence Scale to align with the research context. Specific items include: “I believe the priorities of my employing volunteer service team are quite important,” “My employing volunteer service team provides valuable public services,” and “For me, the mission of my employing volunteer service team is exciting.” Higher scores indicate greater mission valence among participants. In this study, we conducted reliability and validity tests on the Mission Valence Scale. The results showed that the Cronbach’s *α* coefficient for the scale was 0.85, the composite reliability (CR) was 0.853, and the average variance extracted (AVE) was 0.7. These results demonstrate that the Mission Valence Scale has good reliability and validity in this study.

#### 3.2.3 Volunteer motivation scale

Volunteer Motivation Scale developed by Chinese scholar Jiang Weichuan was used. This scale is based on the Volunteer Function Scale created by Clary et al. and includes six dimensions: learning and understanding, career development, value expression, self-improvement, self-protection, and social interaction. Each dimension contains three items (Jiang, 2018). Jiang Weichuan translated and revised each item of the scale in accordance with China’s specific circumstances and contextual expressions, incorporating feedback from experts in volunteer studies and senior managers of volunteer service organizations. The revised Volunteer Motivation Scale was subsequently validated among Chinese volunteers, demonstrating sound reliability and validity, making it suitable for application within this demographic. Specific items include: “Participating in voluntary work has given me a fresh perspective on things,” “Participating in voluntary service broadens my career options,” and “Participating in voluntary work makes me feel important,” among others. The Likert 5-point scale was used, higher scores indicate a greater level of volunteer motivation among participants. In this study, we conducted reliability and validity tests on the Volunteer Motivation Scale. The results showed that the Cronbach’s *α* coefficient for the scale was 0.962, with a composite reliability (CR) of 0.934, and the average variance extracted (AVE) was 0.7. These results demonstrate that the Volunteer Motivation Scale has good reliability and validity in this study.

#### 3.2.4 Work engagement scale

Schaufeli et al. (2002) Work Engagement Short Form Scale was used, comprising three dimensions: vigor, dedication, and focus. Each dimension contains three items, for a total of nine items. To assess the applicability of the Work Engagement Scale in China, the instrument was first rigorously translated by professionals specializing in psychology and English. The back-translated English version was submitted to its principal developers for review, and an initial test version was created based on their feedback. After removing items with insufficient discriminative validity, the final version for measurement was established. Results indicate that all metrics of the Chinese Work Engagement Scale meet psychometric requirements, making it suitable for future domestic research on work engagement (Zhang and Gan, 2005). In this study, “job” is defined as “volunteer service” within the work commitment table to align with the research context. Specific items include: “At my volunteer service, I feel bursting with energy,” “At my volunteer service, I feel strong and vigorous,” and “I am enthusiastic about my volunteer service,” among others. The Likert 7-point scale was used, higher scores indicate greater work engagement among participants. In this study, we conducted reliability and validity tests on the Work Engagement Scale. The results showed that the Cronbach’s *α* coefficient for the questionnaire was 0.944, the composite reliability (CR) was 0.932, and the average variance extracted (AVE) was 0.82. These results demonstrate that the Work Engagement Scale has good reliability and validity in this study.

#### 3.2.5 Team atmosphere scale

This study used the Team Atmosphere Scale developed by Chinese scholar Zhang Kejun, comprising three dimensions—team identity, openness, and interpersonal trust, and a total of 19 items (Zhang, 2009). Zhang Kejun based the scale on existing research frameworks for measuring team atmosphere, identifying interpersonal trust and openness as core dimensions. Through observations of real-world teams, he added a dimension of team identity. The revised scale was validated in both corporate and academic settings, demonstrating strong reliability and validity. Within this team atmosphere scale, the term “team members” is defined as “volunteer team members” to align with the research context. Specific items include: “All volunteer team members are willing to make every effort to achieve the team’s objectives,” “Volunteer team members possess a strong sense of belonging to the team,” and “Volunteer team members all believe they can rely on one another,” among others. In this study, we conducted reliability and validity tests on the Team Atmosphere Scale. The results showed that the Cronbach’s *α* coefficient for the questionnaire was 0.964, the composite reliability (CR) was 0.904 and the average variance extracted (AVE) was 0.76. These results demonstrate that the Team Atmosphere Scale has good reliability and validity in this study.

### 3.3 Statistical processing

This study used AMOS 24.0, SPSS 26.0, and PROCESS for statistical data analysis. The Harman single-factor method and latent method factor analysis were used to test common method bias. Descriptive statistics were used to analyze demographic variables. Independent samples t-tests and one-way ANOVA were used to test demographic differences in work engagement. Pearson correlation analysis was used to examine correlations among servant leadership, mission valence, volunteer motivation, work engagement, and team atmosphere. Mediation and moderation effects were tested using bootstrap methods. *p* < 0.05 indicates statistically significant differences.

### 3.4 Common method bias assessment

The collected data were analyzed using SPSS 26.0 via Harman’s single-factor test. The results revealed six factors with eigenvalues greater than 1, without rotation. The first factor explained 19.15% of the variance, below the 40% critical threshold.

This study further employed latent method factor analysis, adding an additional latent method factor into the five-factor model. After adding this factor, the model fit indices (*χ*^2^/df = 8.48, RMSEA = 0.075, NFI = 0.936, RFI = 0.916, IFI = 0.943, TLI = 0.926, CFI = 0.943) showed no significant improvement compared to the original five-factor model (*χ*^2^/df = 8.8, RMSEA = 0.077, NFI = 0.936, RFI = 0.916, IFI = 0.943, TLI = 0.926, CFI = 0.943) did not show significant improvement (Wen et al., 2018). Therefore, we conclude that there was no significant common method bias in this study.

### 3.5 Confirmatory factor analysis

This study used AMOS to perform confirmatory factor analysis on five-factor, four-factor, three-factor, two-factor, and single-factor models, using RMSEA, CFI, and TLI to assess model fit. Although the chi-square value indicates the degree of fit between sample data and the model, it is highly sensitive to sample size, excessively large samples tend to inflate the chi-square value (Bollen, 1989). Since the sample size in this study reached 1,330 individuals, additional indicators were consulted for evaluation. The results show that the five-factor model achieved ideal fit indices and significantly outperformed other models (RMSEA = 0.077, CFI = 0.933, TLI = 0.922). This indicates significant discriminant validity among servant leadership, volunteer motivation, mission valence, team atmosphere, and work engagement. Detailed results are shown in Table 1.

**Table 1:** Results of validation factor analysis.

Model	*χ*^2^/df	RMSEA	NFI	RFI	IFI	TLI	CFI
Five-factor model	8.8	0.077	0.925	0.913	0.933	0.922	0.933
Four-factor model	19.715	0.119	0.829	0.806	0.837	0.814	0.836
Three-factor model	21.679	0.125	0.809	0.786	0.817	0.794	0.816
Two-factor model	26.159	0.138	0.768	0.742	0.775	0.75	0.775
One-factor model	29.207	0.146	0.74	0.712	0.746	0.719	0.746

The five-factor model is Servant leadership, Volunteer motivation, Mission valence, Work engagement, Team atmosphere.

The four-factor model is Servant leadership, Volunteer motivation, Mission valence, Work engagement + Team atmosphere.

The three-factor model is Servant leadership, Volunteer motivation, Mission valence + Work engagement + Team atmosphere.

The two-factor model is Servant leadership, Volunteer motivation + Mission valence + Work engagement + Team atmosphere.

The single factor model is Servant leadership + Volunteer motivation + Mission valence + Work engagement + Team atmosphere.

## 4 Results

### 4.1 Testing for differences in volunteer work engagement in blood donation services

The results indicate significant differences in volunteer work engagement in blood donation services based on age, educational attainment, frequency of volunteer service, and years of service (*p* < 0.05). No significant gender differences were found in work engagement. See Table 2 for details.

**Table 2:** Demographic differences test.

Item	Number	Percentage (%)	Work engagement		
			Score	*t*/*F*	*p*
Gender				0.709	0.478
Man	430	32.3	38.08 ± 10.1		
Female	900	67.7	37.65 ± 10.31		
Age				11.149	*p* < 0.01
18–27	1,224	92	37.31 ± 10.11		
28–37	23	1.7	40.61 ± 11.71		
38–47	14	1.1	43.64 ± 9.39		
48–57	29	2.2	40.03 ± 10.34		
>57	40	3	47.08 ± 8.31		
Educational attainment				2.849	0.036
Secondary school\high school and below	111	8.4	40.5 ± 11.18		
Specialty	422	31.7	37.54 ± 10.76		
Undergraduate	790	59.4	37.55 ± 9.76		
Postgraduates	7	0.5	36.71 ± 11.18		
Frequency of volunteering				32.862	*p* < 0.01
≤1 time per week	828	62.3	36.21 ± 10.15		
2 times per week	364	27.4	39.46 ± 9.77		
≥3 times per week	138	10.4	42.83 ± 9.69		
Years of volunteer service				13.752	*p* < 0.01
Less than 1 year	769	57.8	36.63 ± 10.37		
1 ≤ 3 years	404	30.4	38.27 ± 9.54		
3–5 years	75	5.6	42.2 ± 9.95		
>5 years	82	6.2	42.23 ± 10.24		

### 4.2 Correlation test

The results show significant positive correlations among servant leadership, mission valence, volunteer motivation, work engagement, and team atmosphere. Detailed findings are presented in Table 3.

**Table 3:** Descriptive statistics and correlation analysis.

Variant	*M*	SD	Servant leadership	Volunteer motivation	Mission valence	Work engagement	Team atmosphere
Servant leadership	3.98	0.64	1				
Volunteer motivation	4.22	0.60	0.686^**^	1			
Mission valence	4.43	0.59	0.552^**^	0.615^**^	1		
Work engagement	4.20	1.14	0.610^**^	0.767^**^	0.562^**^	1	
Team atmosphere	4.37	0.57	0.621^**^	0.652^**^	0.774^**^	0.578^**^	1

** Significant correlation at 0.01 level (two-tailed).

### 4.3 Testing the main effects of the relationship between servant leadership and work engagement

This study used multilevel linear regression to examine the relationship between servant leadership and work engagement. To examine the relationship between servant leadership and work engagement, work engagement was treated as the dependent variable. Regression analyses were conducted by sequentially adding control variables and servant leadership. The results, as shown in Table 4, indicate that servant leadership is associated with higher levels of work engagement (*β* = 0.595, *p* < 0.01). Thus, assume H1 is validated.

**Table 4:** Main effects test.

Predictor variable	Work engagement					
	Model 1			Model 2		
	*β*	*t*	*p*	*β*	*t*	*p*
Age	0.1	2.989	0.003	0.104	3.936	*p* < 0.01
Educational attainment	0.024	0.802	0.423	0.015	0.622	0.534
Frequency of volunteering	0.182	6.586	*p* < 0.01	0.127	5.783	*p* < 0.01
Years of volunteer service	0.087	2.872	0.004	0.078	3.251	0.001
Servant leadership				0.595	28.282	*p* < 0.01
*R* ^2^	0.069			0.42		
*F*	24.677			191.614		

### 4.4 Testing the mediating effects of mission valence and volunteer motivation

Further investigate the mediating effect of volunteer motivation and mission valence on the relationship between servant leadership and work engagement. The results show that servant leadership significantly and positively relates to work engagement, mission valence, and volunteer motivation (*β* = 0.136, 0.546, 0.495, *p* < 0.01). Mission valence positively and significantly predicted volunteer motivation and work engagement (*β* = 0.336, 0.098, *p* < 0.01). Volunteer motivation significantly and positively predicted work engagement (*β* = 0.597, *p* < 0.01). The direct effect of servant leadership on work engagement along with the mediating effects of mission valence and volunteer motivation, were significant. The mediating effect values for mission valence and volunteer motivation were 0.053 and 0.295, respectively, and the serial mediating effect value was 0.11, with bootstrap 95% confidence intervals not containing zero. This indicates that servant leadership not only directly relates to work engagement (*β* = 0.136, *p* < 0.01) but also indirectly relates to work engagement through the mediating effects of volunteer motivation and mission valence (*β* = 0.458, *p* < 0.01). The direct effects and mediating effects accounted for 23 and 77%, respectively. Furthermore, significant differences existed among the mediating effects, with the effect of volunteer motivation being significantly higher than that of mission valence and serial mediation. Therefore H2, H3, and H4 are validated. The specific results are shown in Tables 5, 6.

**Table 5:** Path coefficients.

Predictor variable	Mission valence			Volunteer motivation			Work engagement		
	coeff	se	*t*	coeff	se	*t*	coeff	se	*t*
Age	0.09*	0.03	2.61	−0.03	0.03	−1.03	0.115**	0.03	4.59
Educational attainment	0.05	0.04	1.36	0.03	0.03	0.91	−0.01	0.03	−0.38
Frequency of volunteering	0.07*	0.04	2.05	0.07*	0.03	2.59	0.122**	0.03	4.75
Years of volunteer service	0.03	0.03	0.91	0.03	0.03	1.15	0.066*	0.02	2.99
Servant leadership	0.546**	0.02	23.91	0.495**	0.02	22.42	0.136**	0.02	5.85
Mission valence				0.336**	0.02	15.14	0.098**	0.02	4.52
Volunteer motivation							0.597**	0.03	24.16
*R* ^2^	0.315			0.553			0.64		
*F*	121.8			273.196			335.428		

*p < 0.05, **p < 0.01.

**Table 6:** Mediating effect test.

Path	Effect	SE	LLCI	ULCI	Percentage	Percentage
Total effect	0.595	0.021	0.553	0.636	1	
Direct effect	0.136	0.023	0.091	0.182	0.23	
Total indirect effect	0.458	0.021	0.417	0.501	0.77	1.00
ind1: Servant leadership → Mission valence → Work engagement	0.053	0.014	0.026	0.082		0.12
ind2: Servant leadership → Volunteer motivation → Work engagement	0.295	0.019	0.258	0.333		0.64
ind3: Servant leadership → Mission valence → Volunteer motivation → Work engagement	0.11	0.012	0.088	0.134		0.24
diff1: ind1-ind2	−0.242	0.027	−0.293	−0.188		
diff2: ind1-ind3	−0.056	0.02	−0.097	−0.018		
diff3: ind2-ind3	0.186	0.024	0.14	0.232		

### 4.5 Testing the moderating role of team atmosphere in the serial mediation path from mission valence to volunteer motivation

**Figure 2:**
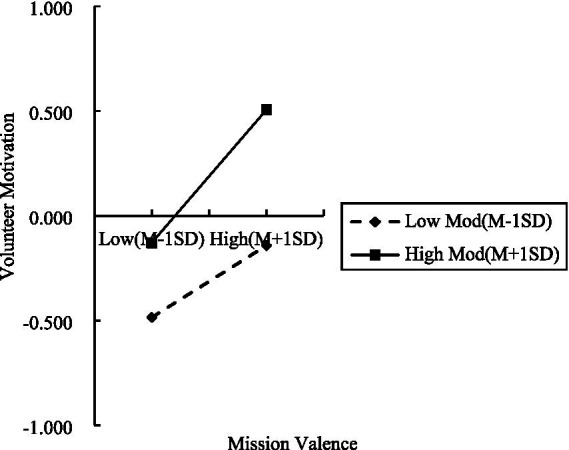
The moderating effect of team atmosphere on the relationship between mission valence and volunteer motivation.

Use the Bootstrap method in the PROCESS macro to examine the moderating effect of team atmosphere. Set the bootstrap to 5,000 iterations with a 95% confidence interval. As shown in Table 7, the interaction term between mission valence and team atmosphere significantly and positively was significantly and positively associated with volunteer motivation (*β* = 0.075, *p* < 0.01). That is, team atmosphere positively moderated the relationship between mission valence to volunteer motivation in the serial mediation model. Therefore, H5 can be considered supported. To further explore the moderating effect of team atmosphere, this study employed simple slope analysis for additional verification. A relationship diagram was plotted showing the correlation between mission valence and volunteer motivation at team atmosphere levels one standard deviation above and below the mean. As shown in Figure 2, at higher levels of team atmosphere, the positive association between mission valence and volunteer motivation is stronger. Therefore, H5 can be further supported.

**Table 7:** Moderating effect of team atmosphere on the relationship between mission valence and volunteer motivation.

Predictor variable	Mission valence			Volunteer motivation			Work engagement		
	coeff	se	*t*	coeff	se	*t*	coeff	se	*t*
Age	0.09**	0.034	2.61	−0.023	0.027	−0.849	0.115**	0.025	4.588
Educational attainment	0.048	0.035	1.364	0.028	0.027	1.037	−0.01	0.025	−0.375
Frequency of volunteering	0.072*	0.035	2.049	0.06*	0.027	2.17	0.122**	0.026	4.751
Years of volunteer service	0.028	0.031	0.905	0.039	0.024	1.646	0.066**	0.022	2.988
Servant leadership	0.546**	0.023	23.908	0.422**	0.023	18.448	0.136**	0.023	5.852
Mission valence				0.249**	0.03	8.448	0.098**	0.022	4.519
Volunteer motivation							0.597**	0.025	24.16
Team atmosphere				0.252**	0.03	8.308			
Mission valence*Team atmosphere				0.075**	0.011	7.099			
*R*	0.561			0.766			0.8		
*R* ^2^	0.315			0.587			0.64		
*F*	121.8			234.927			335.428		

*p < 0.05, **p < 0.01.

### 4.6 Testing the moderated serial mediation effect of team atmosphere

This study employed the Bootstrap method to further examine the serial mediating effects of volunteer motivation and mission valence under varying levels of team atmosphere. The results are presented in Table 8. When team atmosphere is low, the serial mediating effect of servant leadership on work engagement through mission valence and volunteer motivation is weaker (*β* = 0.057, 95% CI = [0.035, 0.082]). When team atmosphere is at a higher level, the serial-mediated effect of servant leadership on work engagement through mission valence and volunteer motivation is stronger (*β* = 0.106, 95% CI = [0.066, 0.153]). Moreover, serial mediating effects exhibit significant differences across varying adjustment levels, with the mediating effect in the high-score group being significantly higher than in the low-score group.

**Table 8:** Moderated mediation effect.

Moderating effect	Team atmosphere	Efficiency value	SE	BootLLC	BootULCI
Moderated serial Mediation model	eff1 (M − 1SD)	0.057	0.012	0.035	0.082
	eff2 (M)	0.081	0.014	0.056	0.111
	eff3 (M + 1SD)	0.106	0.023	0.066	0.153
Comparison of effects	eff2-eff1	0.024	0.012	0.005	0.048
	eff3-eff1	0.049	0.023	0.01	0.096
	eff3-eff2	0.024	0.012	0.005	0.048

## 5 Discussion

This study found that servant leadership is significantly associated with work engagement among blood donation service volunteers, consistent with the findings of Cai et al. (2024). This suggests that servant leadership may be an important factor related to work engagement among volunteers in blood donation services. High work engagement reflects an individual’s positive attitude toward work and dedication. Servant leaders view leadership roles as opportunities to care for and serve others, working within teams to meet volunteers’ needs and support their development. Thus, the exemplary role of servant leadership in helping behaviors may encourage volunteers to imitate and learn, potentially demonstrating greater enthusiasm for their work. At the same time, servant leadership builds mutual trust and respect with volunteers, emphasizes a shared vision among team members, empowers volunteers, provides opportunities for decision-making participation, and enhances volunteers’ autonomy. The findings of Bošjančič et al. suggest that granting volunteers a high degree of autonomy may be associated with greater work enthusiasm (Eva et al., 2018). Additionally, servant leadership provides professional training for volunteers, potentially enhancing their skills, capabilities, and confidence. By communicating with volunteers, servant leadership facilitates timely feedback on their work, helps resolve challenges they face, and offers prompt recognition and appreciation for their contributions. This approach may increases volunteer satisfaction with blood donation services, potentially boosting their motivation.

The results of this study suggest that servant leadership is associated with the work engagement of blood donation service volunteers through mission valence and volunteer motivation. Finkelstein suggests that volunteers may be more likely to continue volunteering when one of their functional motivations is fulfilled. Specifically, blood donation service volunteers under servant leadership are associated with a higher likelihood of perceiving the mission and significance of blood donation volunteering work, which may be associated with fulfilling their value motives, making them more likely to focus on volunteering work. In their study of volunteers, Syamaliah et al. (2022) found that servant leadership was associated with higher volunteer satisfaction and stronger support for the vision and mission communicated to them by the organization. In daily training and volunteer work, servant leadership conveys the social significance of blood donation service work to volunteers through direct or indirect means, helping volunteers understand the importance of blood donation volunteer service, which may be associated with higher sense of mission. The people-centered values of servant leadership emphasize helping members develop, focusing on enhancing blood donation service volunteers’ abilities, increasing their sense of individual value, and aligning personal values with the organization’s mission, which may be associated with engaging in volunteer behaviors consistent with the organization’s mission.

Individuals’ behaviors are driven by their internal will, and those with higher volunteer motivation exhibit a stronger intrinsic drive, which may help maintain cognitive and behavioral consistency, potentially leading to higher volunteer motivation. Previous studies have shown that intrinsic motivation is associated with work engagement (Bao and Zhang, 2005). Blood donation service volunteers who perceived more servant leadership are associated with stronger volunteer motivation. The qualities of a servant leadership, which prioritizes others and dedicates themselves to the public interest may be associated with their members’ volunteer motivation. Over time, through the long-term cultivation of the spirit of service, team members may gradually adopt and learn from the servant leadership values and behaviors focused on serving others. Gagné (2014) noted that fulfilling an individual’s intrinsic needs may help enhance motivation to work based on self-determination. Servant leadership cares about the development and needs of their members, and is willing to provide resources and support. This may be associated with the appeal of participating in blood donation services, stimulating a sense of responsibility and enthusiasm, and potentially enhancing volunteer motivation. Ultimately, this may encourage the long-term commitment of blood donation service volunteers.

This study also found that mission valence and volunteer motivation play a serial mediating role in the relationship between servant leadership and work engagement, with a direct association of mission valence on volunteer motivation, consistent with previous research (Caillier, 2015). This suggests that the higher the level of mission valence among blood donation service volunteers, the stronger their volunteer motivation may be. Servant leadership is not only directly associated with the work status of volunteers, but may also indirectly be related to their work engagement by shaping their value perception and intrinsic motivation toward the mission of the organization. Specifically, individuals with high mission valence may be more drawn to the organization’s mission and vision, better understanding and aligning with its values, which may be associated with an increased willingness to participate in volunteering and stimulating their intrinsic volunteer motivation. Previous research has proposed that nonprofit employees are largely drawn to their work, and that attraction to the mission of their work may be associated with their level of service motivation (Word and Carpenter, 2013). Moreover, servant leadership may be associated with blood donation service volunteers’ motivation to volunteer by increasing their mission valence, which may ultimately strengthen their willingness to commit to the work. When leading blood donation service volunteers, servant leadership may help them understand the deeper value of the organization’s mission by continuously conveying their vision and providing care. This may be associated with enhanced volunteers’ recognition of the mission, their sense of belonging, and their loyalty to the organization. As a result, volunteers may gain a better understanding of their work, and may experience a higher sense of satisfaction and achievement during the service process, which may boost their confidence in achieving the organization’s goals, thus increasing their willingness to participate in blood donation volunteer services. The stronger an individual’s motivation to volunteer, the more proactive, focused, and persistent they may become in their work, which may contribute to improving the efficiency and quality of the service.

This study examined the moderating role of team atmosphere in the relationship between mission valence and volunteer motivation. The results show that team atmosphere is associated with the serial mediation pathway of “servant leadership → mission valence → volunteer motivation → work engagement.” Mission valence is associated with volunteer motivation to varying degrees, depending on the level of team atmosphere. When the team atmosphere is high, volunteers’ perception of the team atmosphere is associated with a stronger facilitative association of mission valence on volunteer motivation; conversely, this association may weaken at lower levels. This suggests that when team members identify with the team’s goals and trust each other, mission valence may be associated with greater volunteer engagement in volunteering. Previous research has shown that teams with higher functioning and a better atmosphere are associated with a stronger connection to the mission and vision of the organization, and with members recognizing and supporting the team’s values and goals (Innocent et al., 2022). In a positive team atmosphere, individuals’ cognition and behavior are more likely to align with others. When team members exhibit strong service spirit and positive service behavior, individuals may align their cognition, adopt similar behaviors, and contribute to maintaining a positive team identity. A positive team atmosphere is associated with emotional support and strengthened volunteers’ sense of belonging through shared goals and values. As the team atmosphere improves, individuals’ identification with the mission of organization may be associated with greater intrinsic motivation to volunteer. This approach is associated with a reinforced understanding of the organization’s mission and greater volunteer motivation, which may be associated with work engagement.

## 6 Conclusion

This study is based on the current social context of high volunteer turnover and insufficient work engagement. It uses social exchange theory as the core theoretical framework, complemented by self-determination theory, AMO theory, and social identity theory to examine the relationship between servant leadership and work engagement as well as its potential mechanisms. The results showed that servant leadership was significantly associated with work engagement among blood donation service volunteers, and was linked to work engagement via mission valence and volunteer motivation. Team atmosphere is significantly associated with the serial mediation pathway from servant leadership to work engagement via mission valence and volunteer motivation.

## 7 Significance and limitations of the study

This study employed a moderated serial mediation model to show that servant leadership enhances blood donation service volunteers’ work engagement, both directly and indirectly, through the serial mediating roles of mission valence and volunteer motivation. Additionally, team atmosphere significantly moderates the relationship between mission valence and volunteer motivation, with a stronger effect when team atmosphere is more positive. This finding extends the research on the factors influencing of blood donation service volunteers’ work engagement and its underlying mechanisms. It also provides empirical evidence for improving the willingness of blood donation service volunteers’ work engagement, offering both theoretical and practical significance.

This study has the following limitations: first, the cross-sectional design used in this study does not allow for the identification of causal relationships between variables, and future research may further adopt a longitudinal design to explore the dynamic relationships between variables more accurately. Second, this study only explored the interactions between servant leadership, mission valence, volunteer motivation and team atmosphere and their effects on work engagement. Future research should further explore the mechanisms of other variables on the work engagement of blood donation service volunteers to deepen understanding and support the sustainable development of unpaid blood donation services.

## Data Availability

The original contributions presented in the study are included in the article/supplementary material, further inquiries can be directed to the corresponding author.
